# Correlation between classification and secondary screw penetration in proximal humeral fractures

**DOI:** 10.1371/journal.pone.0183164

**Published:** 2017-09-06

**Authors:** Qiuke Wang, Yu Zhu, Yifei Liu, Lei Wang, Yunfeng Chen

**Affiliations:** Department of Orthopedic Surgery, Shanghai Jiao Tong University Affiliated Sixth People’s Hospital, Shanghai, P.R., China; Georgia Regents University, UNITED STATES

## Abstract

**Objectives:**

In this study, we investigated the correlation between fracture classification and secondary screw penetration.

**Methods:**

We retrospectively identified 189 patients with displaced proximal humeral fractures treated by ORIF at our hospital between June 2006 and June 2013. All fractures were classified radiographically before surgery and follow-up for least 2 years after surgery was recommended. At each follow-up, radiographs were taken in three orthogonal views to evaluate secondary screw penetration.

**Results:**

The study population consisted of 189 patients. Of these, 70 were male and 119 female, with a mean age of 59.1 years; the mean follow-up time was 28.5 months. Secondary screw penetration occurred in 26 patients. The risk of developing secondary screw penetration was 11.3-fold higher in four-part fractures than two-part fractures (*P* < 0.05), 8.6-fold higher for type C fractures than type A fractures (*P* < 0.05) and 11.0-fold higher for medial hinge disruption group than intact medial hinge group fractures (*P* < 0.05). However there was no difference between three-part fractures and two-part fractures (*P* = 0.374), and between type B and type A fractures (*P* = 0.195). Age, gender, time to surgery and the number of screw in humeral head had no influence on the secondary screw penetration rate (*P* > 0.05).

**Conclusions:**

Patients with four-part fractures, type C fractures and medial hinges disruption are vulnerable to secondary screw penetration. This allows additional precautions to be instituted and measures to be taken as needed.

## Introduction

Proximal humeral fracture accounts for 4–5% of all bone fractures, with the growing elderly population, the frequency is still increasing [1–3]. Roughly 20% of these fractures exhibit an unstable fracture pattern, which should be treated by surgery [4]. Numerous operative techniques have been used for fixing unstable fractures, including Kirschner wires, tension band sutures, intramedullary nails, prosthetic replacement and locking plates. Locking plates, which are considered to be a better choice over other implants in fixing osteoporotic bone, are widely used to treat proximal humeral fractures. However, such treatment is associated with a high rate of complications after surgery, among which screw penetration is thought to be the most frequent one. Screw penetration includes both primary and secondary screw penetration; primary screw penetration occurs during surgery and can be avoided by use of a fluoroscope during surgery, while secondary screw penetration occurs after surgery and the cause is still unclear [5–7]. However, secondary screw penetration is considered to be the most frequent complication after locking plate fixation surgery [8–12]. Patients with secondary screw penetration may experience severe pain and require subsequent revision surgery, which has a significant effect on rehabilitation. Although some studies have been carried out concerning the risk factors affecting secondary screw penetration [13,11,14], there is little information about the correlation between fracture classification and secondary screw penetration.

The aim of our study therefore was to explore whether there are any relationships between preoperative fracture classification and secondary screw penetration after surgery. We studied two widely used fracture classification systems: Neer and AO/OTA system. We hypothesized that certain fracture subtypes, according to the two classification systems, would be associated with a higher risk of screw tip penetration.

## Materials and methods

This study was approved by ethics of committee of Shanghai sixth people's hospital, the approval number is 2016-ky-005. From our hospital database, we identified consecutive patients (n = 238) with a proximal humeral fracture fixed using the locking plate technique between June 2006 and June 2013.

Inclusion criteria were: (I) aged 18 years or above, (II) presence of displaced proximal humeral fracture (unilateral), (III) surgical treatment by ORIF with a PHILOS plate (Synthes, Oberdorf, Switzerland), (IV) more than 2 years follow-up after surgery with complete fellow-up data. Exclusion criteria included pathological fractures, open fractures, multiple fractures, follow-up of <2 years or the presence of other diseases affecting the same upper limb.

Ultimately, 189 individuals remained and made up the study population. All patients’ demographics and regular radiographic examinations, taken preoperatively and at each follow-up visit, were available. Preoperative evaluations included X-rays (anteroposterior and lateral views) and computed tomography (CT) scans. Follow-up monitoring included trauma-series x-rays (true glenoid anteroposterior, transscapular lateral and axillary views) on the first day after surgery and at each fellow-up visit.

Fractures were classified according to the description systems of Neer and AO/OTA by two independent examiners, based on evaluation of the preoperative radiographs. The inter-individual kappa value of the two examiners was caculated. All discordant cases were reevaluated by a third examiner, and the results were assigned by consensus of the three examiners. In the Neer classification system, we divided all fractures into three basic fracture types: two, three, and four-part fractures. We distinguished three basic fracture types according to the AO/OTA classification system (A, B and C). Medial hinge disruption was identified according to preoperative X-rays and CT scan (more than 3 medial hinge fragments [15]).

The trauma-series x-rays at the first day after surgery and each follow-up visit were evaluated by an independent examiner to assess screw penetration.

### Surgical procedure

Patients were operated in the beach chair position under general anesthesia or brachial plexus block anesthesia. A standard deltopectoral approach was used, and tuberosity fragments were reduced indirectly with the use of non-absorbable sutures passing through the rotator cuff tendons, then the humeral head was reduced. After all fracture fragments were reduced, Kirschner wires were used for provisional stabilization. Then the locking plate was placed lateral to the bicipital groove. Screws were placed into the humeral head after measuring the depth of the drilling holes, taking care that the screw tips remained 5 mm from the subchondral bone. After that, shaft screws were placed bicortically. Finally, intraoperative fluoroscopy was required to ensure anatomic reduction and check that all proximal locking screws were placed within the humeral head. In some cases, patients with serious osteoporosis or bony defects were treated with an allograft. All surgeries were finished by three experienced attending doctors (28 surgeries were completed by Yu Zhu, 65 surgeries were completed by Lei Wang and 96 surgeries were completed by Yunfeng Chen).

After surgery, a similar rehabilitation protocol was recommended to all patients. Each patient’s affected arm was suspended in a sling for 4 weeks postoperatively. Then patients were allowed to perform a passive range of exercises for 4 weeks, after which, patients started to take active exercises. All patients were recommended follow-up at 4 weeks, 12 weeks, 6 months, 1 year and 2 years after surgery.

### Statistical analysis

Statistical analysis was performed using the statistical program SPSS version 20.0 (IBM Corp., Armonk, NY, USA). We defined age, sex, time to surgery, the number of screw in humeral head, medial hinge integrity and fracture type as the independent variables, secondary screw penetration as the dependent variable. In order to exclude the influence of confounders, multiple factor and non-condition logistic regressions were performed to analyze the correlation between the independent and dependent variables. Results were considered significant when *P* < 0.05, OR and 95%CI were calculated for these results.

## Results

**Fig 1:**
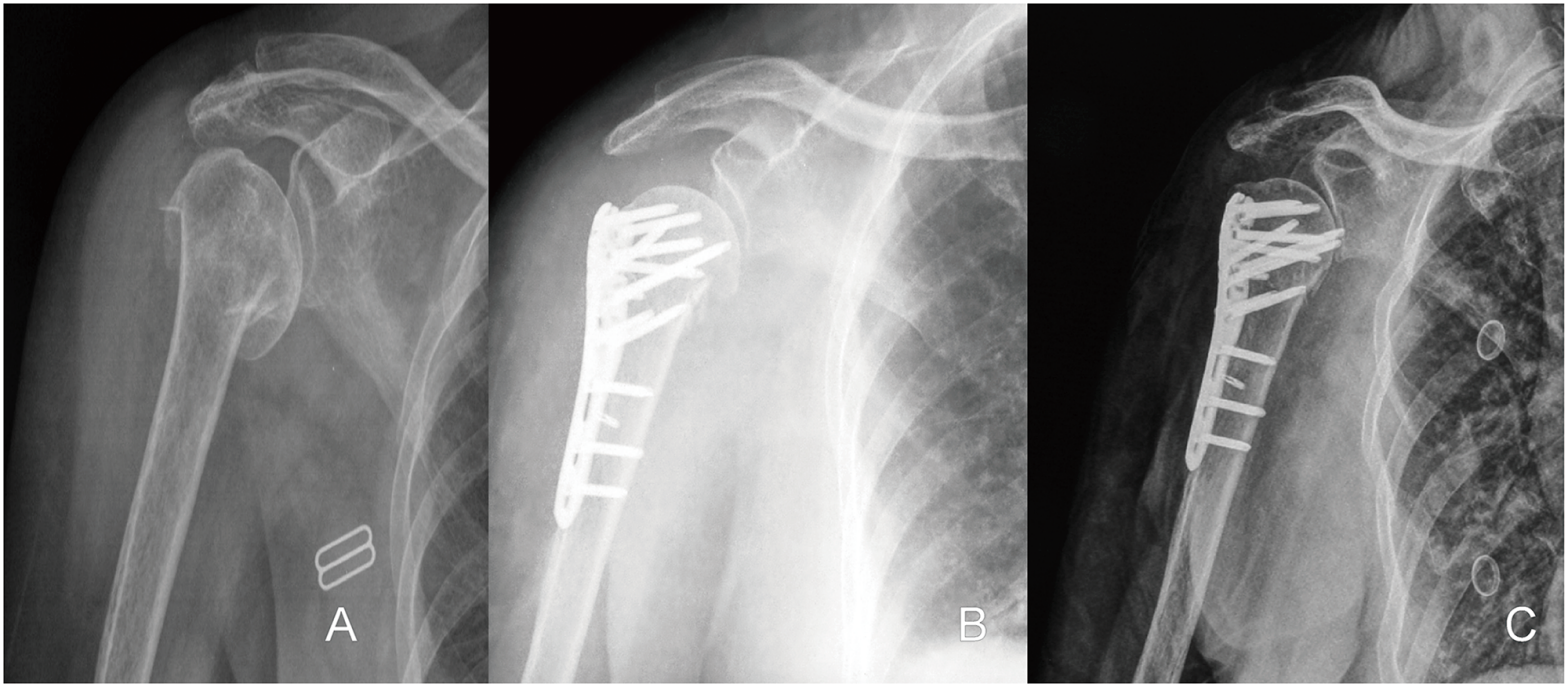
**A**: Anteroposterior shoulder radiograph of an 81-year-old woman who suffered a car accident in which her right shoulder impacted the ground, sustaining a 4-part (AO type C) proximal humeral fracture. **B**: the first day after surgery showing all screws within the humeral head. **C**: Two months postoperatively, screws penetrating the joint.

Of the 238 patients with a displaced proximal humeral fracture fixed with a proximal humeral locking plate in our hospital from June 2006 to June 2013, 189 patients ultimately met the inclusion and exclusion criteria and made up the study population. The study population comprised 70 males (37.0%) and 119 females (63.0%) with a mean age of 59.1 years (range 18–91), and mean time to surgery was 4.3 days (range 2–15 days). With a minimum follow-up time of 2 years, 180 fractures (89.9%) healed while 19 patients suffered non-union, and eight patients suffered humeral head osteonecrosis (4.2%). The mean fellow-up time was 28.5 months (range 24–60 months). The number of screws inserted into the humeral head ranged from 5 to 9 (average 5.8), two calcar screws were placed in all patients except seven patients who suffered isolated greater tuberosity fracture. Secondary screw penetration occurred in 26 patients (13.8%), in whom penetration of the screw through bone could be seen on X-rays of the shoulder (Fig 1). Interestingly, in the 8 patients who suffered humeral head necrosis, 5 patients were complicated with secondary screw penetration and 2 of the 5 patients underwent hemiarthroplasty surgery. Of all secondary screw penetration patients, 14 patients who had secondary screw penetration with severe shoulder pain underwent surgery to remove the screws, while the rest accepted conservative treatment.

**Fig 2:**
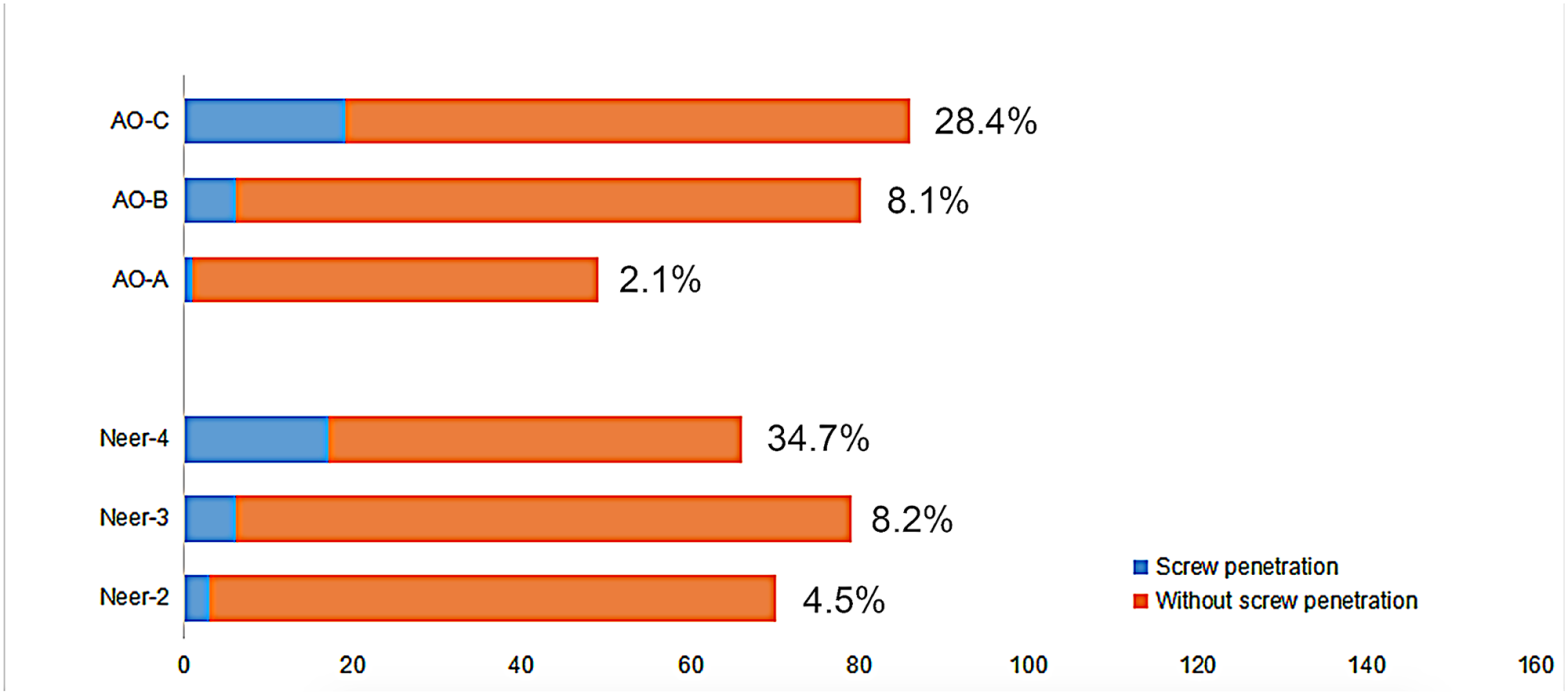
Screw penetration rate in the three fracture classification systems.

Eventually 189 fractures were classified. In the Neer system, 67 (35.4%) were classified as two-part fractures, 73 (38.6%) as three-part fractures and 49 (25.9%) as four-part fractures, while in the AO/OTA system, 48 (25.4%) were type A fractures, 74 (39.2%) were type B fractures and 67 (35.4%) were type C fractures. Fig 2 shows the secondary screw penetration rate according to fracture type of the two fracture classification systems.

In our study, according to the Neer system, the risk of developing secondary screw penetration was 11.3-fold higher in four-part fractures than in two-part fractures (*P* = 0.001, OR = 11.333, 95% CI 3.093–41.530); however, there was no difference between three-part fractures and two-part fractures (*P* = 0.374), the inter-individual kappa value was 0.593. Similarly, in the AO system, the risk of developing secondary screw penetration was 18.6-fold higher in type C fractures than type A fractures (*P* = 0.005, OR = 18.604, 95% CI 2.393–144.618), while the difference between type B and type A was not significant (*P* = 0.195), the inter-individual kappa value was 0.610. In terms of medial hinge integrity, the difference between the medial hinge disruption group and intact medial hinge group was significant (*P* = 0.001, OR = 11.018, 95% CI 4.043–30.029). While age, gender, time to surgery and the number of screw in humeral head had no influence on the secondary screw penetration rate (*P* > 0.05). All patients’ relevant data are available in S1 Fig.

## Discussion

With the increasing use of locking plates in the treatment of proximal humeral fractures, especially in osteoporotic bone, many complications occur postoperatively, such as humeral head malunion or nonunion, humeral head avascular necrosis, screw penetration, osteoarthritis, and infection. Secondary screw penetration, however, is thought to be the most frequent complication after locking plate fixation surgery. According to previous studies, the incidence of secondary screw penetration is 0 to 29% [8–12], and Gardner *et al*. [11] reported the highest rate (29%, 5/17) due to lack of medial support after surgery. However, Owsley *et al*. [14] found that in their research, secondary screw penetration occurred in 12 out of 53 patients (23%), while in an older population (>60 years of age), secondary screw penetration occurred in nine of the 21 patients (43%), indicating that the rate of screw penetration is associated with patient age. In our study, secondary screw penetration occurred in 26 patients (13.8%), and there was no correlation between secondary screw penetration and age. Egol *et al*. [16] found that patients who experienced secondary screw penetration were on average 6 years older than those who did not, but the difference was not significant. As we all know, as age increases, bone density decreases, resulting in more complex fractures with the same degree of damage power, and in a study by Owsley *et al*. [14] a high rate of screw penetration was found in patients older than sixty years who had a three- or four-part fracture, so we believe that there could be a confounder between age, fracture type and secondary screw penetration. Recently, Boesmueller *et al*. [13] studied 154 patients to identify the risk factors for complications in proximal humeral fractures, and found that there were statistically significant correlations between secondary screw penetration and age; however, screw penetration is unrelated to fracture type according to the Neer and AO classification systems. It was disappointed that the medial hinge was not involved in their research, which is considered as an essential risk factor for screw penetration [11,17]. In this study, the OR of the medial hinge disruption was 11 compared with intact medial hinge.

**Fig 3:**
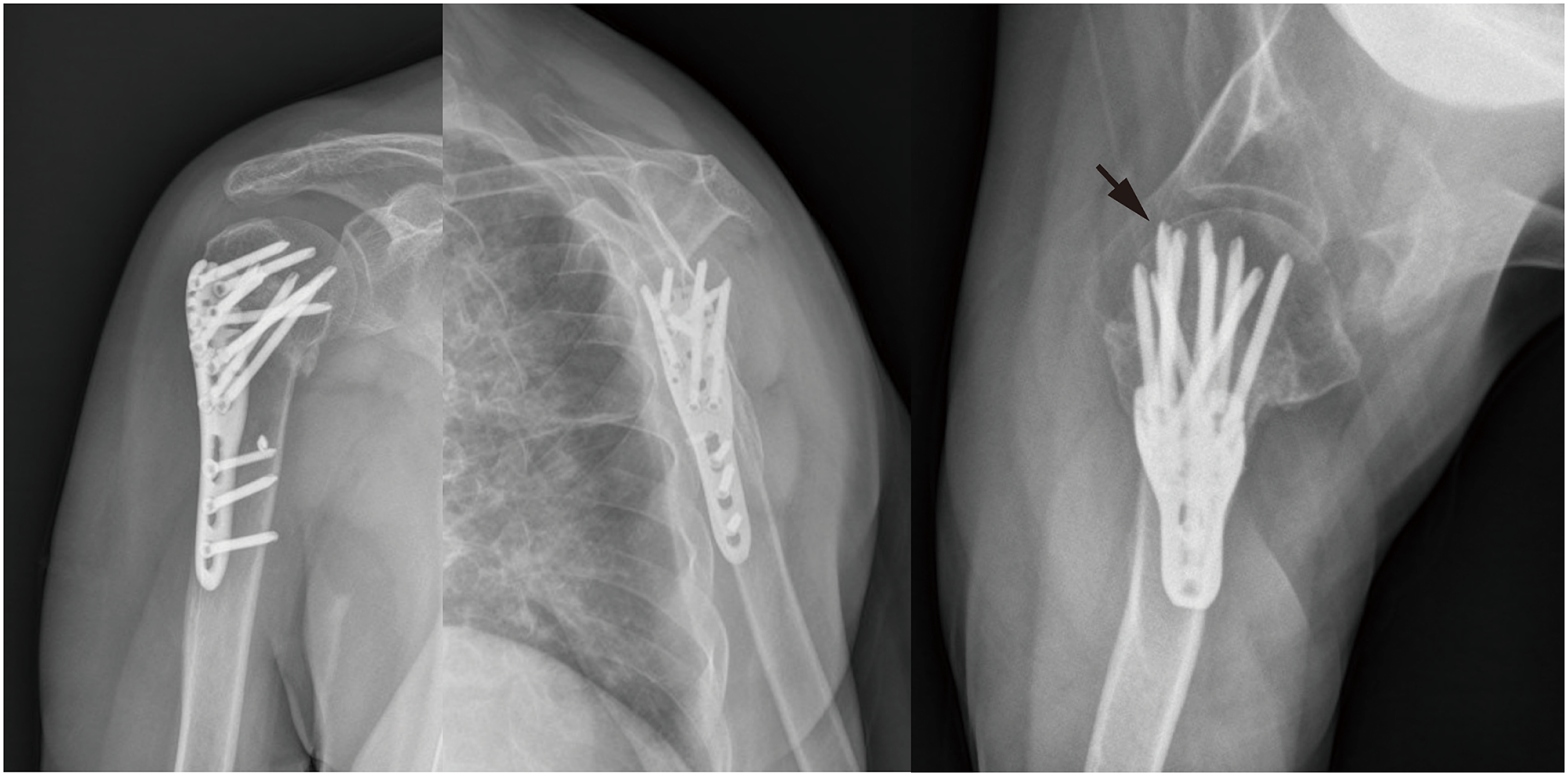
Trauma-series x-rays of a 75-year-old woman at the follow-up of three months after surgery, the black arrow pointed at the screw penetrating the joint while it was missed on the true glenoid anteroposterior and transscapular lateral radiographs.

We used trauma-series x-rays to identify the secondary screw penetration. Three orthogonal views, especially axillary view, had a high sensitivity of detection [18]. Screw penetrations missed on anteroposterior and lateral views might be identified on axillary view (Fig 3). However, axillary view fluoroscopy (abduction 30°) could be very painful for fresh fractures, so only anteroposterior and lateral views were conducted before surgery in this study. Moreover, 19 of 238 patients couldn’t stand the pain of axillary view fluoroscopy on the first day after surgery were excluded, because the first day fluoroscopy was essential for excluding primarily screw penetration.

We proved our hypothesis that there could be a correlation between the complexity of a fracture and secondary screw penetration. In the Neer system, the risk of developing secondary screw penetration was 11.3-fold higher in four-part fractures than in two-part fractures. However, there was no difference between three-part fractures and two-part fractures; the reason for this may be that in our study most three-part fractures were the type involving the greater tuberosity and anatomical neck (60/73), in which the greater tuberosity could be stably fixed because of compression of the laterally-placed plate. In the AO system, the risk of developing screw penetration was 18.6-fold higher in type C fractures than in type A fractures, while the difference between type B and type A was not significant. As we all know that the blood supply of the humeral head is likely to be damaged in type C fractures, which may cause humeral head necrosis. In this study, 8 patients suffered humeral head necrosis and 5 of them complicated with secondary screw penetration, suggesting that there may be a correlation between humeral head necrosis and secondary screw penetration. In a retrospective analysis by Egol *et al*. [16] they found the same thing, in that two patients who developed avascular necrosis of the humeral head also had screw penetration into the glenohumeral joint.

We found no correlation between patients’ demographics (age and gender) and secondary screw penetration. In addition, early intervention was not a good choice for preventing secondary screw penetration because time to surgery had no influence on the secondary screw penetration rate. The average number of screws we put in humeral head was 5.8 (5–9), including two calcar screws, however, this did not affect the secondary screw penetration rate. Erhardt *et al*. [19] advised putting five or more screws (especially including calcar screws) in the humeral head, which can reduce the risk of secondary screw penetration, so our study may have hidden the correlation between screws less than 5 and more than 5 in humeral head.

Given that we believe that complexity of the fracture is related to secondary screw penetration, some measures to enhance the stability of fracture fixation can be taken during the surgery to decrease the rate of secondary screw penetration. As mentioned above, insertion of more than 5 screws, including calcar screws, in the humeral head is recommended. Secondary, Gradl *et al*. [20]and Egol *et al*. [16] reported the same result: that locking plate augmented with calcium phosphate cement could reduce the rate of secondary screw penetration. Thirdly, in some studies, bone grafting has been found to reduce the incidence of screw penetration markedly after surgery [21,22], which can be explained if it is considered that the bone graft helps reduce fracture fragments and makes up the function of the disrupted medial hinge by offering medial support. In our cohort, three people accepted treatment with a bone allograft, and interestingly, all avoided secondary screw penetration. Finally, in patients with high-risk fracture types or medial hinge disruption, other operative techniques should be considered.

There are some limitations to our study: (I) the retrospective nature of the study. (II) Three surgeons performed the operations in our study. Although they each received the same surgical training and had agreed on surgical techniques, personal differences can still not be avoided. (III) Fracture classification bias may exist due to the limited reliability of the two systems, and the kappa values were 0.593 and 0.610 in this study.

## Conclusions

In conclusion, we have identified some correlation between fracture classification and secondary screw penetration. Patients with four-part fractures, type C fractures and medial hinges disruption are vulnerable to secondary screw penetration, while age, sex, time to surgery and the number of screw in humeral head have no influence on the secondary screw penetration rate. Consequently, surgeons should evaluate the proximal humerus fractures they treat and use this to estimate the risk of secondary screw penetration. This allows additional precautions to be instituted and measures to be taken as needed.

## Supporting information

S1 Fig All patients’ relevant data. (XLSX)
